# Unveiling and addressing implementation barriers to routine immunization in the peri-urban slums of Karachi, Pakistan: a mixed-methods study

**DOI:** 10.1186/s12961-021-00691-4

**Published:** 2021-08-11

**Authors:** Amna Tanweer Yazdani, Ameer Muhammad, Muhammad Imran Nisar, Uzma Khan, Yasir Shafiq

**Affiliations:** 1 VITAL Pakistan Trust, Karachi, Pakistan; 2 Department of Pediatrics and Child Health, Aga Khan University, Karachi, Pakistan

**Keywords:** Demand-side, Supply-side, Barriers, Immunization, Coverage, Peri-urban, Slums, Implementation, Integrated, Digital

## Abstract

**Background:**

Great disparities in immunization coverage exist in Pakistan between urban and rural areas. However, coverage estimates for large peri-urban slums in Sindh are largely unknown and implementation challenges remain unexplored. This study explores key supply- and demand-side immunization barriers in peri-urban slums, as well as strategies to address them. It also assesses immunization coverage in the target slums.

**Methods:**

Conducted in four peri-urban slums in Karachi, this mixed-methods study consists of a baseline cross-sectional coverage survey of a representative sample of 840 caregivers of children aged 12–23 months, and 155 in-depth interviews (IDIs) through purposive sampling of respondents (caregivers, community influencers and immunization staff). After identifying the barriers, a further six IDIs were then conducted with immunization policy-makers and policy influencers to determine strategies to address these barriers, resulting in the development of an original validated implementation framework for immunization in peri-urban slums. A thematic analysis approach was applied to qualitative data.

**Results:**

The survey revealed 49% of children were fully vaccinated, 43% were partially vaccinated and 8% were unvaccinated. Demand-side immunization barriers included household barriers, lack of knowledge and awareness, misconceptions and fears regarding vaccines and social and religious barriers. Supply-side barriers included underperformance of staff, inefficient utilization of funds, unreliable immunization and household data and interference of polio campaigns with immunization. The implementation framework’s policy recommendations to address these barriers include: (1) improved human resource management; (2) staff training on counselling; (3) re-allocation of funds towards incentives, outreach, salaries and infrastructure; (4) a digital platform integrating birth registry and vaccination tracking systems for monitoring and reporting by frontline staff; (5) use of digital platform for immunization targets and generating dose reminders; and (6) mutual sharing of resources and data between the immunization, Lady Health Worker and polio programmes for improved coverage.

**Conclusions:**

The implementation framework is underpinned by the study of uncharted immunization barriers in complex peri-urban slums, and can be used by implementers in Pakistan and other developing countries to improve immunization programmes in limited-resource settings, with possible application at a larger scale. In particular, a digital platform integrating vaccination tracking and birth registry data can be expanded for nationwide use.

## Background

Immunization is one of the most cost-effective life-saving investments, proven to thwart vaccine-preventable diseases and avert 2–3 million deaths annually [1]. However, despite its many successes, global immunization coverage remains at 85% without any significant change in recent years, with 1.5 million deaths still occurring annually due to vaccine-preventable diseases [2].

The rate of immunization in Pakistan is still suboptimal, with only 66% of children aged 12–23 months being fully immunized [3]. Great disparities in immunization coverage exist between the provinces, and between urban and rural areas. In Sindh, the second most populous province of Pakistan, the overall coverage is 49%—with 37% coverage in rural areas as compared to 63% in Sindh’s urban areas [3]. However, the coverage estimates for large peri-urban slums are conspicuously absent in various demographic and health surveys. These rapidly growing peri-urban slums come with their unique set of challenges that remain largely unexplored.

Health system barriers in relation to the implementation of the Expanded Programme on Immunization (EPI) in Pakistan include problems with programme financing, governance, service delivery, human resources (HR), information systems and supplies and vaccines [4]. Demand-side barriers in Pakistan include a low awareness and knowledge of immunization, concerns and misperceptions about vaccines, belief in local remedies and religious beliefs [4].

The supply- and demand-side barriers that the EPI in Pakistan faces in the context of peri-urban settings are not widely explored. However, in the absence of true population estimation and clear evidence of immunization, accurate targets cannot be set; in addition, the accuracy of reported administrative coverage of immunization in these settings becomes questionable. The hurdles related to poor immunization uptake in peri-urban slums need to be studied in greater depth in order to address these barriers within the existing EPI programme in limited resource settings. Adoption of policy-backed solutions is especially crucial in the context of peri-urban slums with their high childhood mortality rates. Therefore, a strong need for research was identified with the objective of not only exploring key supply- and demand-side immunization barriers in peri-urban slums, but also of unveiling strategies to address them. In addition, this study assessed childhood vaccination status in the target slums to identify pockets of poor vaccination coverage, and to identify respondents for barrier analysis.

## Methods

The study was conducted in four peri-urban slums (Rehri Goth, Ibrahim Hyderi, Ali Akbar Shah Goth and Bhains Colony) along Karachi’s coastal belt. In all four sites, the Department of Pediatrics and Child Health of the Aga Khan University (AKU) has been working closely with VITAL Pakistan Trust (VPT) on maternal and child health interventions. Primary Health Centers (PHCs) are being run at each site, alongside a long-standing demographic surveillance system. All pregnant women and newborns are registered under the system.

This study applied a mix of quantitative and qualitative approaches to explore immunization coverage and implementation barriers, as well as solutions to address them. Qualitative tools are frequently utilized by studies investigating barriers to the implementation of health policies and services due to the richness of the resulting data and its ability to help inform new strategies and interventions. The study was conducted in two phases. During Phase 1, a baseline cross-sectional coverage survey and qualitative in-depth interviews (IDIs) were conducted.

The survey was conducted from June to September 2017 to document the vaccination coverage status of a representative sample of children aged 12–23 months, as well as to identify caregivers for interviews. A close-ended questionnaire was developed, translated into Urdu and pretested. A standard technique, lot quality assurance sampling (LQAS), was used to randomly select 210 households at each site using the demographic surveillance system data, resulting in a total of 840 children aged 12–23 months.

The survey was administered to 840 caregivers during household visits by research staff after obtaining written informed consent. A trained Senior Research Assistant and a locally hired Community Health Worker (CHW) collected the data during each interview, which lasted approximately 30 min. Survey data was analyzed using SPSS statistical software (IBM Corp., Armonk, NY, USA).

Additionally in Phase 1 of data collection, 155 IDIs were conducted with different categories of respondents (Table 1).

**Table 1:** Categories and number of respondents for Phase 1 in-depth interviews

Category of respondents	Number (*n*)
Caregivers	122^a^
Community influencers^b^	17
Lady Health Workers	7
Senior Town Health Management or EPI officials	5
EPI vaccinators	2
Nongovernmental organization mobilizers and vaccinators	2
Total	155

*EPI *Expanded Programme on Immunization,* NGO* nongovernmental organization

^a^72 caregivers refused immunization services and 50 caregivers accepted immunization services

^b^Community influencers included two key informants: spiritual healer, social workers, midwives, family physicians, a principal of an Islamic seminary school, political party representatives, community heads, elderly members of the community and union council members

Phase 1 IDIs were conducted from June 2017 to April 2018 through purposive sampling of respondents (including caregivers identified through the survey who either refused or accepted immunization services) to explore demand- and supply-side immunization barriers. A broad sampling frame and purposive sampling ensured that a wide variety of participants were approached.

Interviews were conducted by a multilingual team, whose composition differed from that of the survey team, comprising an experienced qualitative interviewer assisted by a CHW. Open-ended, semi-structured interview guides consisting of discussion areas and probes were developed in English and translated into Urdu for use, followed by pretesting. After following established guidelines for obtaining written informed consent, the interviews were conducted in local languages at the particpants’ homes, with each interview approximately 45 min in length. All interviews were audio-recorded with the respondent’s permission, and the interviewer completed a verbatim transcript in Roman Urdu script within 1 week of the interview. The transcripts were cross-checked by two senior researchers for consistency before a translator translated them to English. Both quantitative and qualitative data sets were de-identified using case identifiers and stored on devices that only the study team could access.

Phase 2 of research, conducted from June to July 2018, consisted of qualitative data collection based on a structured IDI guide that incorporated Phase 1 findings. The interviews were conducted by the investigators ATY and YS in the form of consultative meetings with policy-makers and policy influencers, with a majority of the interviews taking place in the capital city of Islamabad at the respondents’ offices. During the interviews, Phase 1 findings were disseminated and strategies to address those barriers were determined. The six Phase 2 respondents included top-level officials from Federal EPI, Town Health Management and donor agencies and a representative of a leading immunization civil society organization (CSO). Thematic analysis of Phase 2 data resulted in the development of an original validated implementation framework for immunization in peri-urban slums by the study investigators, featuring policy recommendations for the barriers identified in Phase 1. The framework was deemed to be validated through its endorsement by individuals with considerable influence on immunization policy.

The study utilized an inductive thematic analysis methodological approach, with qualitative analysis undertaken by an experienced analyst. Open coding of the transcripts was followed by axial coding using NVivo 11 Plus software (QSR International, Doncaster, Australia). Nodes were ascribed to patterns across the dataset, then studied for linkages and collapsed into themes with subsequent refinement. To enhance the trustworthiness of the data, the coded data and themes were reviewed by a second senior researcher, and team meetings were held on a weekly basis throughout the study to discuss data collection, transcription, emergent themes and coding. With analysis occurring alongside data collection, the latter continued until data saturation was observed and no additional insights were being provided by the data.

Ethical approval for all study procedures was obtained from the Research Ethics Review Committees of the WHO and the AKU. Voluntary and informed consent from participants was obtained free from any coercion and after thorough explanation of the study procedures.

## Results

### Baseline vaccination coverage survey

**Fig. 1:**
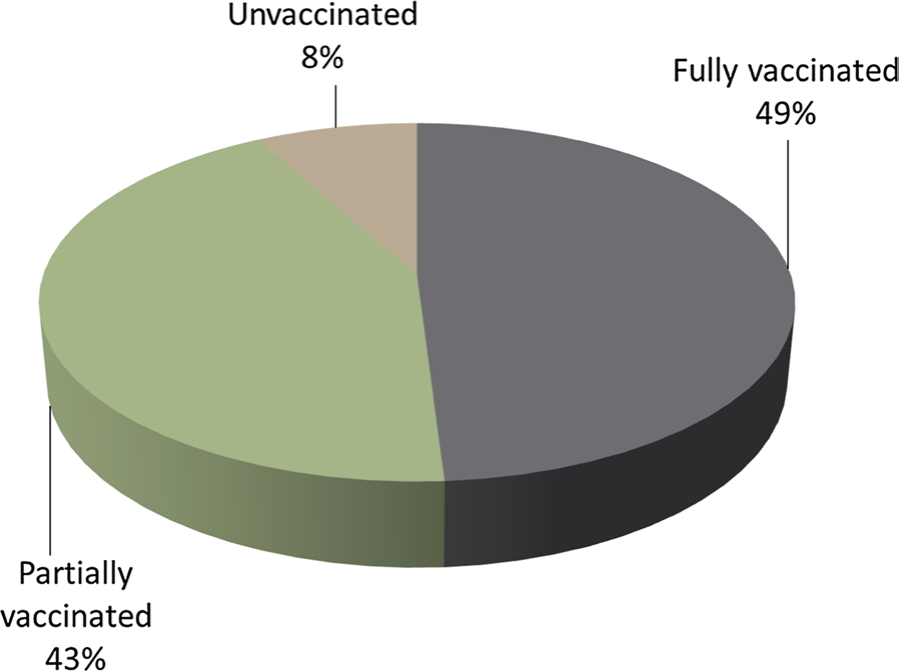
Immunization coverage among children aged 12–23 months in the study areas (*n* = 840)

**Fig. 2:**
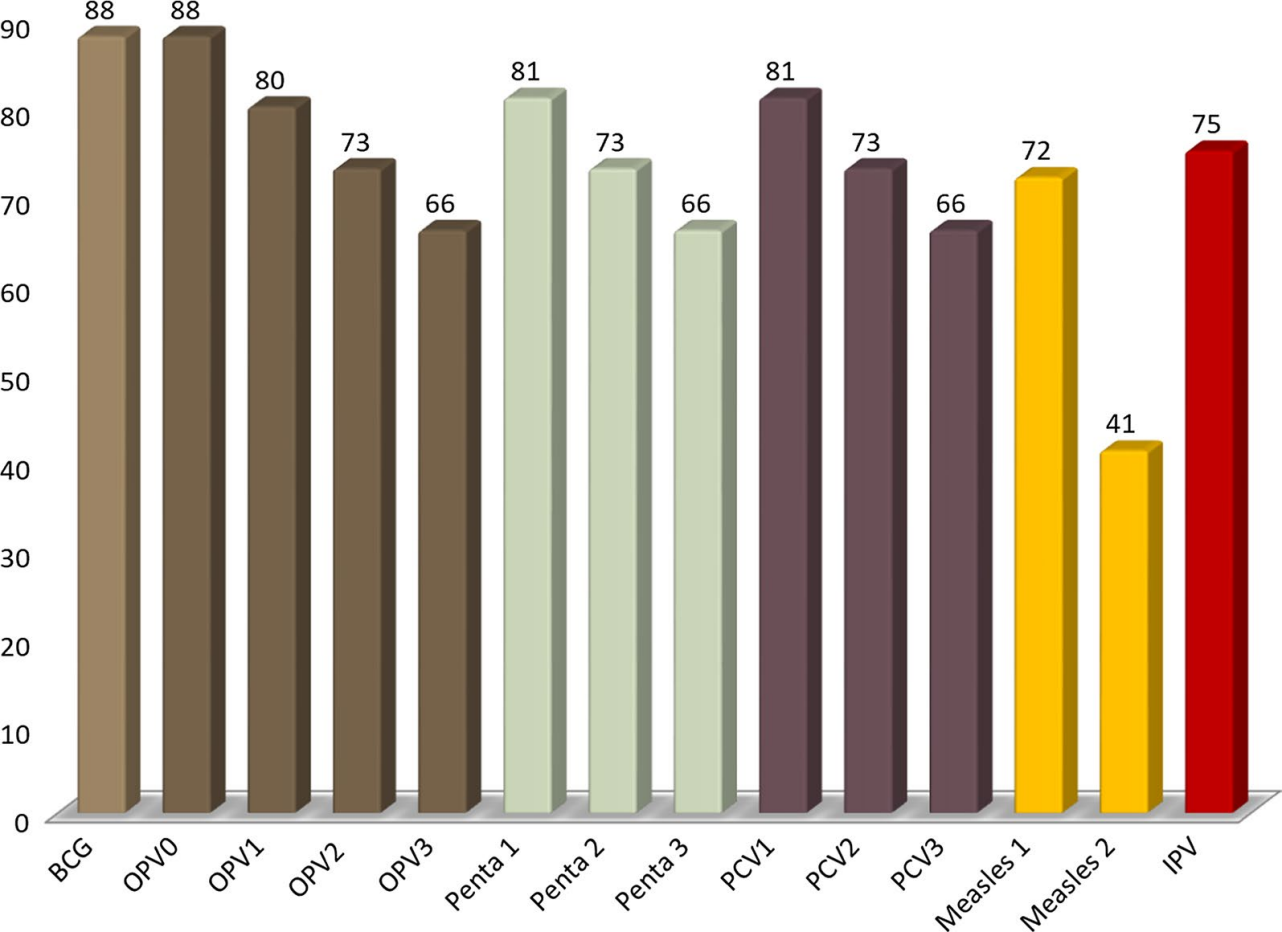
Immunization coverage by antigen in percentage among children aged 12–23 months in the study areas (*n* = 840)

The vaccination coverage of the target slums is shown in Fig. 1. Antigen-wise coverage is given in Fig. 2, which shows that the highest vaccination rates are for the Bacillus Calmette-Guérin (BCG) and oral polio vaccine given at birth (OPV0) and the lowest coverage is for the second dose of measles vaccine (Measles 2).

**Fig. 3:**
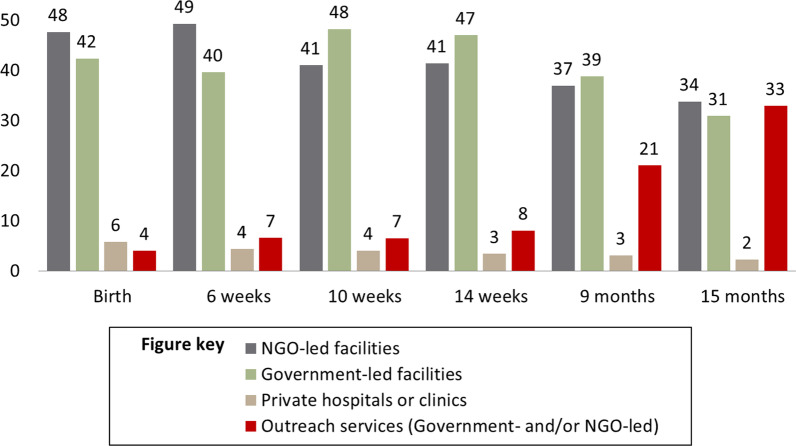
Immunization service utilization in percentage at different points in the vaccination schedule (*n* = 840)

The survey also revealed that only 51% of respondents who received any vaccine dose reported retention of the EPI card. Additionally, scars from the BCG vaccine were reported as present in 72% of cases. Moreover, as shown in Fig. 3, a greater utilization of outreach services by families was seen for doses later in the vaccination schedule.

### Demand-side barriers to immunization

#### Household barriers

Permission for immunization was frequently withheld by the main decision-maker (in most cases, the child’s father or a family elder). Additionally, women had restricted mobility, many only permitted to leave their homes with a companion. Several respondents complained that household duties prevented them from seeking immunization services, and that family members were not supportive.

According to the father of a partially vaccinated child, “*If a husband does not permit the child to get vaccinated, the wife will not go. In this area, none of the husbands approve of vaccination…only when a child gets ill then she has permission to take the child to the hospital, but with another woman*.”

#### Lack of knowledge and awareness

 Many caregivers had little knowledge about vaccine-preventable diseases and were not aware of the benefits of immunization or the consequences of forgoing it.

A mother of an unvaccinated child remarked: “*I didn’t do anything to protect my daughter against measles as I thought that she should get them, as people say that it is better for children to have measles early on…the symptoms are not as serious in kids as they are in adults*.”

#### Myths, misconceptions and fears

Common beliefs that immunization caused sterility, early puberty, illnesses, fever and disabilities discouraged caregivers. Some respondents also believed that providers used expired or impure vaccines, while several others perceived initial vaccine doses to be adequate for protection against all diseases.

According to a father of an unvaccinated child, “*We get scared because these vaccines are imported…it’s obvious that as the vaccines are imported from other countries, they definitely want to halt our generations. These vaccines also cause boys and girls to reach puberty quickly and decrease men’s ability to reproduce*.”

#### Social and religious barriers

Stigma from relatives and the community prevented many caregivers from accepting immunization. Several respondents also believed that immunization was religiously forbidden. Many mentioned that religious or social influencers had prohibited immunization.

According to a popular spiritual healer of the community, “*Our supreme protector is God…I have also gotten sick but I never took any medicine, I only drink water that has been blessed with the name of God…if you stay in a state of ablution you’ll stay protected…there is no need for routine immunization for protection*.”

### Supply-side implementation barriers to immunization

#### Underperformance and negligence of immunization center staff

Reports of forceful or dismissive behaviour of staff members towards caregivers were plentiful. A lack of commitment to tasks was also reported to stem from confusion about the division of roles and responsibilities. A lack of monitoring mechanisms and accountability was also described. Frontline staff, such as vaccinators, were reported to underperform in terms of limited outreach engagement and frequent absences. Staff members also mentioned receiving inadequate training on community mobilization and counselling.

According to a Lady Health Worker (LHW), “*Someone should be present at the centre daily…the vaccinator is not present there, he would only come for two days out of the week and would be absent for the rest. Parents get confused…they don’t want to come back again, after observing the condition of our centre*.”

#### Inefficient allocation and utilization of funds

There were several complaints regarding limited funds for outreach activities, such as inadequate provision of motorcycles and petroleum, oil and lubricants (POL) for vaccinators. Additionally, there were no performance-based incentives and lack of direct salary payments for frontline staff, with complaints of funds being withheld by senior management. Further, vaccination centres were said to have subpar infrastructure and maintenance.

According to a Vaccination Supervisor, “*When all the work is done by the vaccinators but the funds are going to one person (Town Health Officer), problems arise…we can make people work only when we facilitate them and provide money, but that’s not the system here*.”

#### Unreliable and underutilized immunization coverage and household data

Data collected by frontline staff and record-keeping by LHWs was criticized for incompleteness, whereby unimmunized children are not documented using personal identifiers and hence cannot be approached during outreach activities. Additionally, the lack of a birth registry and community line-listing to facilitate vaccinators and support outreach activities was discerned, as well as the absence of an electronic system to track coverage.

According to a Senior EPI Supervisor, “*If we talk about data then the national programmes we have, like LHW programme, are not authentic…according to the list, there were five children in a house but when I visited I found just one child there, so the data they took is either incorrect or reshuffling occurred*.”

#### Interference of polio campaigns with routine immunization

There were several claims that an exclusive emphasis on polio vaccination by frontline staff was leading caregivers to choose it over adherence to routine immunization. Additionally, exhaustion of resources and staff due to polio campaigns was reported. Some respondents shared that when doctors who draw caregivers to immunization centers are redirected to polio campaigns, caregivers stop visiting the centres to get their children vaccinated.

According to a nongovernmental organization (NGO) vaccinator, “*LHWs…their main focus is polio…LHWs take us to the houses and if we encounter a family that refuses vaccinations, they (LHWs) say ‘leave them…don’t even counsel them, don’t pressurize them.’ Instead of helping us, they side with the mothers, saying ‘…because of you they’ll even refuse the polio vaccine as their kids will run a fever due to the vaccination you give them*.”

### Phase 2 analysis: the six-step validated implementation framework for immunization in peri-urban slums

**Fig. 4:**
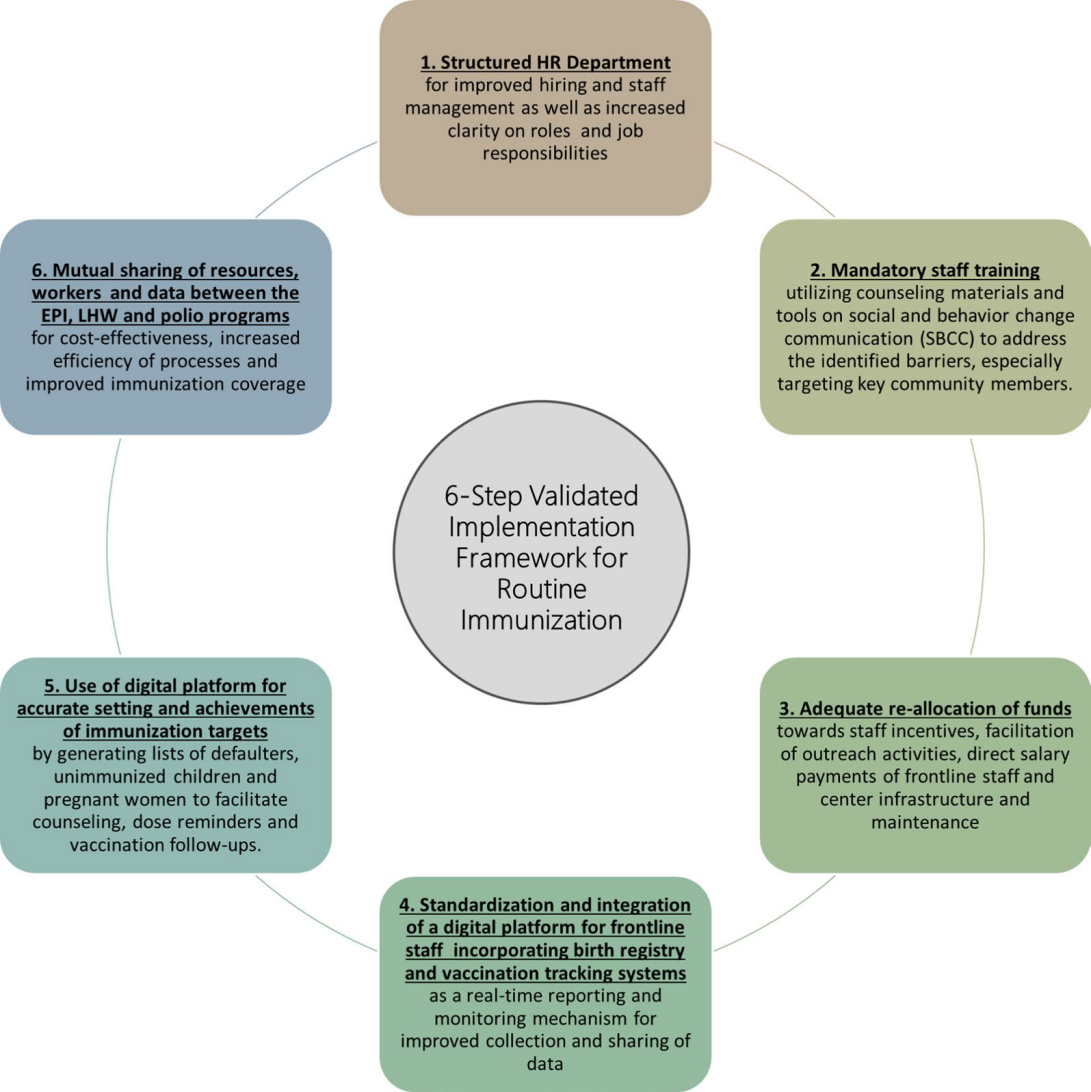
The six-step validated implementation framework for routine immunization

The Phase 2 analysis led to the development of an immunization implementation framework for peri-urban slums. The policy recommendations contained in the framework to address the aforementioned barriers include: (1) a structured HR department for improved immunization staff management; (2) staff training on counselling, and social and behavior change communication (SBCC); (3) re-allocation of funds towards staff incentives, POL and vehicles for outreach activities, direct salary payment of frontline staff and centre infrastructure; (4) a digital platform for frontline staff integrating birth registry and vaccination tracking systems as a real-time monitoring and reporting mechanism; (5) use of the digital platform for setting accurate immunization targets as well as for generating dose reminders; and (6) mutual sharing of resources, workers and data between the EPI, LHW and polio programmes for cost-effectiveness and improved immunization coverage. The recommendations are detailed in Fig. 4.

## Discussion

This study found low vaccination coverage in the target slums of Pakistan, and a similar situation can be seen in the slum areas of neighbouring countries [5, 6]. This low coverage is greatly influenced by supply- and demand-side barriers [7]. Quality immunization services and proper implementation are key to effective vaccine delivery programmes [7].

Performance of the EPI staff, such as vaccinators, is instrumental for improving vaccination coverage [8]. In this study, EPI staff were frequently reported to underperform and lack clarity on job responsibilities. This can have a detrimental impact on immunization coverage. In many vaccination centres, high staff absenteeism and unavailability is a major contributor towards low immunization coverage [9, 10]. This study also linked the poor performance of frontline workers to the unavailability of monitoring mechanisms for the EPI. This is a major weakness of public health interventions in developing countries, where workers are not held accountable for underperforming [9, 11]. Additionally, outreach vaccination services that are crucial in targeting unvaccinated children in conservative communities were found to be inadequate; similarly, outreach services are conducted at a very small scale in many slum communities [12].

Efficient use and transfer of the allocated immunization budget has remained an issue with countries like Pakistan [9, 13]. The study found that the flow of money from provincial EPIs to districts and towns to front line staff is quite problematic, due to money “leakages” along the way. There is also the issue of inadequate incentives for vaccinators and provisions to facilitate outreach work. Discouragingly, workers who pay out-of-pocket for outreach work expenses are not reimbursed [9, 13, 14].

Additionally, the quality of EPI data is questionable, with major issues in reporting [15, 16]. Discrepancies between administrative coverage, vaccinator-reported coverage and survey coverage is a persistent problem [17, 18]. In Pakistan, where the birth registry system is not available, the issue of an underreported denominator presents a huge problem in estimating true coverage. This is exacerbated by a lack of coordination between LHWs and vaccinators, which inflicts a major threat on the programme [19].

For urban slums with their contextual challenges, the current system has failed to address the issues in a robust way, especially by not utilizing technology as a major leverage point for intervention. Redundant paper-based registers are still used in many parts of the developing world, resulting in poor quality data.

Demand-side barriers can be a major contributor to the failure of a programme despite its interventions [6]. At the household level, women’s lack of autonomy is a major immunization barrier in many countries like Pakistan, where men and religious authorities heavily influence decision-making [20]. Additionally, the woman’s restricted mobility prevents her from going to unfamiliar areas or where cultural barriers exist [21].

In slum populations with diverse social behaviors, religious beliefs, cultural boundaries and languages, it is challenging for health providers to create awareness or execute change in the community [21]. Moreover, beliefs, myths and fears related to vaccines are varied across ethnic groups. In urban slum areas, the population mix also leads to proliferation of conservative belief systems to other ethnic groups, creating a system failure where health workers are untrained to deal with such scenarios [21]. Strategies which are effective for one ethnic group may not work for another.

Additionally, lack of awareness regarding vaccination schedules and an inadequate reminder system are major hurdles at both the community and supply-side level [22]. The low level of EPI card retention by vaccine recipients in the survey signifies that adherence to EPI schedules cannot be determined through the card alone.

Slum residents may also believe that the first vaccine dose is sufficient for protection against all diseases [5]. Alongside qualitative findings, the study’s survey findings also corroborate this, with the highest coverage observed for BCG and OPV0 vaccines, and a decline observed for later vaccines with the lowest coverage for Measles 2.

A systematic review of studies on immunization coverage in different urban poor and slum contexts [23] found that immunization services for slums should be designed in accordance with the local context and provided in consultation with slum residents, along with the minimizing of barriers to access, such as geographic and social distance; these are factors that the implementation framework has tried to incorporate. Modest but well-designed interventions can have a major impact on coverage; for example, a study conducted in Karachi showed that in peri-urban areas, the effect of a simple educational intervention (designed for low-literate parents) improved DPT-3 (third dose of diphtheria/pertussis/tetanus vaccine)/Hepatitis B vaccine completion rates by 39% [24].

It is important to understand the need for health interventions designed to particularly increase immunization coverage in peri-urban slums. While total urban coverage levels may be higher than those in rural areas, these numbers mask gaps as central or capital areas are better covered than other urban areas, leaving peri-urban slums with the same or even worse coverage as compared to rural areas [22]. A study on childhood vaccination in Kenya found that children living in urban informal settlements are the most disadvantaged subgroup and do not benefit from the urban advantage of health services [25].

While it is not the primary focus of the current study to analyse the execution of the implementation framework, some challenges to its implementation can certainly be anticipated and prepared for. A significant obstacle could be the endorsement of the framework by policy-makers and influencers within the government and EPI programmes, as well as CSOs, international NGOs and donor organizations. This can be mitigated through the development of a policy brief with actionable recommendations and the sharing of this brief with relevant stakeholders, as well as dissemination meetings for advocacy of the framework. Additional steps to mediate challenges can include public and private sector partnerships that facilitate app development and technical support for the digital platform, sharing of birth registry and health service tracking data and sharing of resources, including training and professional development support for staff. Furthermore, cross-cutting working groups that involve all major stakeholders will help in streamlining processes and cutting down parallel efforts and duplication of interventions with increased resource sharing and transparency. Nevertheless, further research, including a pre- and postintervention analysis of the suggested recommendations, will be useful in assessing the impact of the six-step implementation framework and its implementation challenges.

## Strengths and limitations

The generalizability and causal inference of qualitative data are limited by nature, however this study’s strength lies in trying to comprehensively capture immunization barriers in complex limited-resource settings and present strategies to address them through the involvement of policy implementers and influencers. The study provides tangible new ways to offset implementation barriers in the complicated context of Karachi’s peri-urban slums. To the authors’ knowledge, the present study is the first of its kind to adopt a two-stage approach to qualitative data collection for the purpose of investigating and addressing implementation barriers in Pakistan.

Additionally, the findings may be applicable to other slum populations in developing countries with a possibility for application at a larger scale, particularly the recommendation for a digital platform integrating vaccination tracking and birth registry data.

## Conclusion

The primary outcome of this study is an implementation framework that addresses uncharted barriers in the context of complex peri-urban slums with recommendations for innovating health interventions. Instead of replicating ineffective models that do not address the particular needs of slums, the policy recommendations embedded within the framework have been driven from the ground up. They encompass the perspectives of the community and frontline staff, as well as the expertise of programme implementers.

This framework can be used by implementers in Pakistan and other developing countries to improve the execution and impact of immunization programmes in the limited-resource context of slums, with a possibility for application at a larger scale. The principal policy recommendation of a digital platform integrating vaccination tracking, birth registry and line-listing data is one that can be expanded for nationwide use.

## Data Availability

The datasets generated and/or analysed during the current study are not publicly available due to privacy concerns.

## Abbreviations

AKU: Aga Khan University
CHW: Community Health Worker
CSO: Civil society organization
EPI: Expanded Programme on Immunization
HR: Human resources
IDIs: In-depth interviews
LHW: Lady Health Worker
LQAS: Lot quality assurance sampling
NGO: Nongovernmental organization
PHC: Primary Health Centre
POL: Petroleum, oil and lubricants
SBCC: Social and behavior change communication
VPT: VITAL Pakistan Trust
WHO: World Health Organization
